# *ARS2* Plays Diverse Roles in DNA Damage Response, Fungal Development, and Pathogenesis in the Plant Pathogenic Fungus *Fusarium graminearum*

**DOI:** 10.3389/fmicb.2019.02326

**Published:** 2019-10-15

**Authors:** Duc-Cuong Bui, Jung-Eun Kim, Jiyoung Shin, Jae Yun Lim, Gyung Ja Choi, Yin-Won Lee, Jeong-Ah Seo, Hokyoung Son

**Affiliations:** ^1^Department of Agricultural Biotechnology and Research Institute of Agriculture and Life Sciences, Seoul National University, Seoul, South Korea; ^2^School of Systems Biomedical Science, Soongsil University, Seoul, South Korea; ^3^Therapeutic & Biotechnology Division, Center for Eco-Friendly New Materials, Korea Research Institute of Chemical Technology, Daejeon, South Korea

**Keywords:** *Fusarium graminearum*, arsenite-resistance protein 2, DNA damage response, fungal reproduction, fungal virulence

## Abstract

Arsenite-resistance protein 2 (Ars2) is an important nuclear protein involved in various RNA metabolisms in animals and plants, but no Ars2 ortholog has been studied in filamentous fungi. Although it is an essential gene in most model eukaryotes, *FgARS2* null mutants were viable in the plant pathogenic fungus *Fusarium graminearum*. The deletion of *FgARS2* resulted in pleiotropic defects in various fungal developmental processes. *Fgars2* mutants were irregular in nuclear division, and conidial germination was significantly retarded, causing the fungus to manifest its hypersensitive phenotypes under DNA damage stress. While *FgARS2* deletion caused abnormal morphologies of ascospores and defective ascospore discharge, our data revealed that *FgARS2* was not closely involved in small-non-coding RNA production in *F. graminearum*. The dominant nuclear localization of *Fg*Ars2-green fluorescent proteins (GFP) and abnormal nuclear division in *FgARS2* deletion mutant implicated that *Fg*Ars2 functions in the nucleus. Intriguingly, we found that *Fg*Ars2 established a robust physical interaction with the cap binding complex (CBC) to form a tertiary complex CBC-Ars2 (CBCA), and disruption of any CBCA complex subunit drastically attenuated the virulence of *F. graminearum*. The results of the study indicate that Ars2 regulates fungal development, stress response, and pathogenesis via interaction with CBC in *F. graminearum* and provide a novel insight into understanding of the biological functions of Ars2 in filamentous fungi.

Duc-Cuong Bui, Department of Pathology and Microbiology, University of Nebraska Medical Center, Omaha, NE, United States

## Introduction

Genome maintenance is a constant concern for the identity and function of eukaryotic cells. However, DNA is continuously damaged by endogenous and environmental agents, posing a serious threat to the faithful transmission of genetic information (Rouse and Jackson, 2002). Defects caused by un-repaired or mis-repaired DNA damages result in genomic instability, cell cycle arrest, and lethality, underlining the importance of these processes in the cell and whole organism (Zhou and Elledge, 2000). Therefore, organisms have evolved efficient DNA damage response (DDR) mechanisms that regulate cell-cycle-specific targets to maintain cellular viability and genome stability.

In response to DNA damage, a complex of kinase-based signaling networks is activated to arrest the cell cycle and allow time for DNA repair, which prevents further progression through the cell cycle as long as the lesions persist (Jackson and Bartek, 2009). While extensive investigations in model yeasts provide current critical insights into its nature, the DDR mechanism of filamentous fungi possesses several distinctive features, such as cell death, because of their multicellular fungal hyphae (Goldman et al., 2002). In addition, the post-transcriptional regulation by non-coding RNAs and RNA-binding proteins was recently discovered to be involved in the DDR (Boucas et al., 2012), indicating that cells utilize diverse and flexible cellular regulatory mechanisms for genotoxic responses.

The arsenite-resistance protein 2 (ARS2) is an essential nuclear protein involved in various nuclear RNA metabolisms including 3′ end processing, mRNA or small non-coding RNA (sRNA) biogenesis and export, and degradation (Gruber et al., 2009; Sabin et al., 2009; Giacometti et al., 2017). ARS2 interacts directly with the assembled CBP20/80 cap binding complex (CBC) to form CBC-Ars2 (CBCA) and, consequently, ARS2 frequently determines the fates of transcripts by bridging the CBCA to the appropriate RNA processing machinery (Andersen et al., 2013). Meanwhile, *Arabidopsis thaliana* ARS2 ortholog SERRATE has been shown to bind directly to Dicer-like 1 (AtDCL1), the plant RNase III enzyme responsible for pre-miRNA processing (Iwata et al., 2013). Similarly, ARS2 and the CBC are required at upstream steps in both *Drosophila* and mammalian RNA silencing pathways (Gruber et al., 2009; Sabin et al., 2009). In *Drosophila*, *Dm*Ars2 physically interacts with Dicer-2 and is required for its antiviral properties regulated by siRNA-mediated silencing; therefore, the loss of *DmARS2* increases the susceptibility to RNA viruses in flies (Sabin et al., 2009).

ARS2 is highly conserved, and its orthologs are found in the genomes of most eukaryotic organisms, except *Saccharomyces cerevisiae* (Wilson et al., 2008). Decades of genetic studies revealed the necessity of *ARS2* in fission yeast, plants, insects, and mammals (Golling et al., 2002; Oh et al., 2003; Amsterdam et al., 2004; Lobbes et al., 2006; Wilson et al., 2008; Kim et al., 2010). In addition, it is likely that *ARS2* plays a critical role as a developmental regulator in cell proliferation. In plants, mutation of the *A. thaliana ARS2* ortholog resulted in pleiotropic abnormalities during shoot development (Clarke et al., 1999; Prigge and Wagner, 2001), while the overexpression of *AtSE* caused increased leaf production and earlier flowering (Prigge and Wagner, 2001). In human cells, *HsARS2* expression is linked to the proliferative state of the cell, and a conditional *HsARS2* knockout in hematopoietic tissues leads to decreased cellularity in bone marrow (Gruber et al., 2009). However, the precise biological and biochemical functions of *ARS2* and the CBC in pathogenic fungi are largely unknown.

The ascomycete *Fusarium graminearum* is an important plant pathogen causing Fusarium head blight (FHB) in cereal crops worldwide (Sutton, 1982; Goswami and Kistler, 2004). The serious consequences of *F. graminearum* infection are not only the losses of yield and quality, but contamination of the grains by mycotoxins, such as trichothecenes and zearalenone, that threaten human and animal health (Desjardins, 2006). Although *F. graminearum* reproduces both sexual (ascospores) and asexual (conidia) spores, ascospores are thought to have essential roles as primary inocula (Sutton, 1982; Guenther and Trail, 2005; Leslie and Summerell, 2006). In addition, the initial structures or associated hyphae of the perithecia also function as survival structures for overwintering, indicating its indispensable part in the fungal life cycle (Sutton, 1982; Guenther and Trail, 2005). Since previous biological and chemical strategies to control FHB have not been effective in preventing disease outbreaks, a comprehensive understanding of the cellular and molecular mechanisms of fungal development and pathogenesis as well as the host stress/defense responses is necessary to develop effective and sustainable methods to manage FHB (Goswami and Kistler, 2004).

Our previous studies identified sixteen putative transcription factors (TFs) involved in DDR and further demonstrated that cell cycle regulation and DDR were closely related to sexual reproduction and virulence in *F. graminearum* (Son et al., 2011c; Min et al., 2014; Son et al., 2016). In this study, we attempted to functionally analyze another gene involved in DDR, *FgARS2*, using cellular, genetic, and biochemical approaches. *FgARS2* was not the essential gene in *F. graminearum*, in contrast to the lethality of its orthologs in model eukaryotes. Therefore, the objectives of the study were to uncover the genetic and biological functions of *FgARS2* in DDR, fungal development, and pathogenesis in *F. graminearum*. Our findings shed light on the novel role of the CBCA complex and DDR in the fungal pathogenesis. To our knowledge, this study is the first to characterize the *ARS2* ortholog in filamentous fungi and provides new insights into its diverse roles in fungal developmental processes and pathogenesis.

## Materials and Methods

### Fungal Strains and Media

The *F. graminearum* wild-type strain Z-3639 (Bowden and Leslie, 1999) and the mutants used in this study are listed in Supplementary Table S1. Standard laboratory methods and culture media for *Fusarium* species were used (Leslie and Summerell, 2006). For fungal sporulation, the conidia of all strains were induced on yeast malt agar (YMA) (Harris, 2005) or in carboxymethyl cellulose (CMC) medium (Cappellini and Peterson, 1965). The growth temperature for the fungal strains was 25°C. The wild-type and transgenic strains were stored as mycelia and conidia in 20% glycerol at −80°C.

### Nucleic Acid Manipulation and Genetic Modifications

The genomic DNA was extracted following the standard protocol (Leslie and Summerell, 2006). Restriction endonuclease digestion, agarose gel electrophoresis, gel blotting, and DNA blot hybridization were performed using standard techniques (Sambrook and Russell, 2001). The PCR primers (Supplementary Table S2) used in this study were synthesized by an oligonucleotide synthesis facility (Bionics, Seoul, South Korea).

To complement the *Fgars2* deletion mutants, 5′ and 3′ flanking region including open reading frame (ORF) of the *FgARS2* allele was amplified using the FgARS2-5F com/FgARS2-3N com primer pair from *F. graminearum* strain Z-3639 (Supplementary Table S2). The hygromycin resistance cassette (*HYG*) was amplified from the pBCATPH vector using the pBCATPH-comp 5′For/pBCATPH-comp 3′Rev primer pair (Min et al., 2012). The resulting amplicons were fused via double-joint (DJ) PCR as previously described (Yu et al., 2004). The final PCR constructs were transformed into the *Fgars2* deletion mutants as described previously (Han et al., 2007).

To generate the *FgCBP80* and *FgCBP20* deletion mutants, the 5′- and 3′-flanking regions of each gene and a geneticin resistance cassette (*GEN*) were amplified from Z-3639 and pII99, respectively, and were fused by a second round DJ PCR. The subsequent procedures for the third round of PCR and transformation were the same as those used for complementation of the *FgARS2* gene.

To construct mutants overexpressing a green fluorescent protein (*GFP*)-tagged *FgARS2*, the 5′-flanking region of the *FgARS2* and *FgARS2* ORFs were amplified using primer pairs FgARS2-5F OEG/FgARS2-5R OEG and FgARS2-3F OEG/FgARS2-3R OEG, respectively. A *GEN-P*_*EF*1α_ -*GFP* fragment carrying elongation factor 1α promoter (*P*_*EF*1α_) of *F.verticillioides* followed by the *GFP* ORF was amplified from pSKGEN (Lee et al., 2011) with the primers Neo-for new and eGFP-P1. Three amplicons were fused as described above. A final product was amplified using the nested primers FgARS2-5N OEG and FgARS2-3N OEG.

### Stem-Loop Reverse Transcription- and Quantitative Real-Time (qRT)-PCR

To detect small RNAs, stem-loop RT-PCR analyses of siRNAs and milRNAs were performed as previously described (Chen et al., 2005; Varkonyi-Gasic et al., 2007). Briefly, RNAs highly enriched for small RNA species (<200 nt) were isolated using a *mir*Vana^TM^ miRNA isolation kit (Invitrogen, Carlsbad, CA, United States). Each 100 ng of RNA samples and the stem-loop RT primers that hybridized to the small RNA molecules were used for the reverse transcription reactions, followed by amplification of RT products using miRNA-specific forward primer and the universal reverse primer from 25 to 30 cycles (Supplementary Table S2). The amplification products were visualized on 3% agarose gels.

Total RNA for qRT-PCR was extracted from mycelia that were grown in CM for 3 days or perithecia after 7 days of sexual induction using an Easy-Spin total RNA extraction kit (iNtRON Biotechnology, Seongnam, South Korea). First-strand cDNA was synthesized with a SuperScript III First-Strand Synthesis System (Invitrogen) using oligo(dT)_20_ according to the manufacturer’s instructions. qRT-PCR was performed using an iQ SYBR Green Master Mix (Bio-Rad, Hercules, CA, United States) and a CFX real-time PCR system (Bio-Rad). The endogenous housekeeping gene ubiquitin C-terminal hydrolase (*FgUBH1*; FGSG_01231) was used for normalization (Kim and Yun, 2011). The PCR assays were repeated three times with two biological replicates. The transcript level relative to that of the housekeeping gene was expressed as 2^–ΔΔ*CT*^ (Livak and Schmittgen, 2001).

### Sexual Development and Virulence Tests

Mycelia grown on carrot agar for 5 days were mock-fertilized with sterile 2.5% Tween 60 solution to induce sexual reproduction as previously described (Leslie and Summerell, 2006). Eight days after induction, the perithecia from each strain were dissected in a drop of 15% glycerol, and the asci rosettes within the perithecia were observed under a DE/Axio Image A1 microscope (Carl Zeiss, Oberkochen, Germany). For ascospore discharged observation, a semicircular agar block (11 mm in diameter) that was covered with mature perithecia was placed on a coverslip and incubated in the chamber for 24 h (Trail et al., 2005).

A virulence test of the fungal strains was performed using the wheat cultivar Eunpamil as previously described (Son et al., 2011a). Briefly, 10 μL of a conidial suspension (1 × 10^5^ conidia mL^–1^) of each strain was point-inoculated onto a spikelet of the wheat head at early anthesis. Inoculated plants were incubated in a humidified chamber for 3 days and subsequently transferred to a greenhouse. After 21 days, the number of spikelets showing disease symptoms was counted.

### Microscopic Observation

Microscopic observation was performed using a DE/Axio Imager A1 microscope (Carl Zeiss) equipped with the filter set 38HE (excitation 470/40; emission 525/50) for *GFP* and the filter set 15 (excitation 546/12; emission 590) for red fluorescent protein (*RFP*).

Wheat heads inoculated with the *GFP* expressing strains were sampled 6 days after inoculation. Freehand longitudinal sectioned across the center of the spikelets were prepared using a clean scalpel (Baldwin et al., 2010). Sectioned wheat heads were observed under reflected light and *GFP*-fluorescent light (470-nm excitation and 525-nm emission wavelength filters) using a SteREOLumar V12 microscope (Carl Zeiss).

### Yeast Two-Hybrid Assay

A yeast two-hybrid assay was conducted using the DUALhunter System (Dualsystems Biotech, Zurich, Switzerland) according to the manufacturer’s instructions. To construct plasmids, the coding sequence of each tested gene was amplified from the cDNA of Z-3639 using primers with a *Sfi*I restriction enzyme site (Supplementary Table S2). Full-length *FgARS2* and *FgCBP20* cDNAs were cloned into the pDHB1 vector, a Cub-based bait vector, while full-length *FgCBP80* and *FgCBP20* cDNAs were cloned into pRN3-N, a NubG-based prey vector.

*Saccharomyces cerevisiae* strain NMY51 (MATa his3Δ200 trp1-901 leu2-3, 112 ade2 LYS2:(lexAop)_4_-HIS3 ura3:(lexAop)_8_-lacZ ade2:(lexAop)_8_-ADE2 GAL4) was grown in YPAD medium (1% Bacto yeast extract, 2% Bacto peptone, 2% glucose, and 0.004% adenine sulfate). Bait and prey plasmids were co-transformed using the lithium acetate method (Gietz and Schiestl, 2007). Transformants were selected after 3–4 days of growth at 30°C on synthetic dextrose (SD) medium lacking histidine, leucine, tryptophan, and adenine (SD-Leu/-Trp, SD-Leu/-Trp/-His, and SD-Leu/-Trp/-His/-Ade). Empty vector and pDL2-Alg5 (–) were included as negative controls, and pAI-Alg5 (+) was included as a positive control.

## Results

### *FgARS2* Is Required for Vegetative Growth and Maintenance of Cell Cycle Progression

**FIGURE 1:**
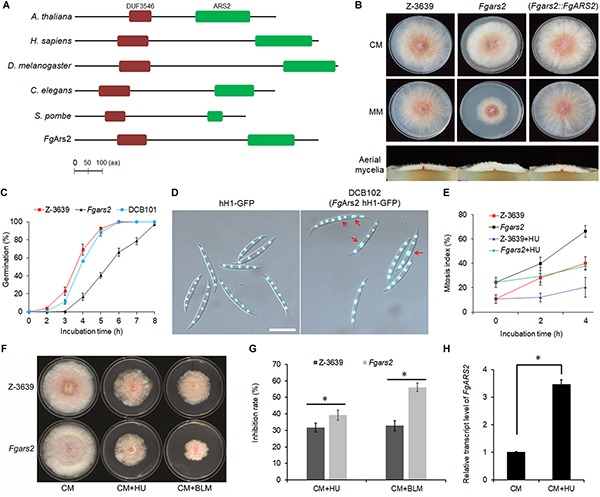
Requirements of *F. graminearum* arsenite-resistance protein 2 for vegetative growth, DNA damage response, and maintenance of cell cycle progression. **(A)** Domain structures of *Fg*Ars2 along with those of representative orthologs created using the InterPro database (http://www.ebi.ac.uk/interpro/) and the HMMPham database (http://pfam.sanger.ac.uk/). DUF3546, domain DUF3546; ARS2, ARS2 domain. The amino acid sequences of Ars2 orthologs *A. thaliana* (NP_565635), *Homo sapiens* (AAI09118), *Drosophila melanogaster* (NP_724455), *Caenorhabditis elegans* (CCD68572.1), *Schizosaccharomyces pombe* (NP_595488), *F. graminearum* (Gene ID: FGSG_01106) are shown. **(B)** Mycelial growth of *F. graminearum* strains on CM and MM. Pictures were taken 4 days after inoculation. **(C)** Germination rate. Percentage of conidial germination in CM. **(D)** Nuclei formation in conidia. Conidia of both strains were inoculated onto yeast malt agar (YMA) for 48 h. Histone H1 was tagged with *GFP* to visualize the nuclei in both wild-type and *Fgars2* mutant strains. DIC and fluorescent protein images were merged. Scale bar = 10 μm. **(E)** Mitosis assay. Germlings bearing cells with two or more nuclei for 4 h incubation with and without 100 mM HU were scored. One hundred conidia were assessed for each strain with three biological replicates. **(F)** Sensitivity of *Fgars2* to DNA-damaging agents, bleomycin (BLM, 20 mU/mL) and hydroxyurea (HU, 10 mM). Pictures were taken 5 days after inoculation. **(G)** Mycelial growth inhibition rate obtained from F. The percentage of the mycelial radial growth inhibition was calculated using the following equation: [(*C*–*N*)/*C*] × 100, where *C* is colony diameter of the control (CM), and *N* is that of treatments (CM + BLM or CM + HU). **(H)** Expression profile of the *FgARS2* gene in the *F. graminearum* wild-type strain Z-3639 during HU treatment. Transcript levels were analyzed via qRT-PCR on mycelia grown for 24 h in CM with and without 100 mM HU for 20 min. The expression of *FgARS2* without HU was arbitrarily set to 1. The asterisks indicate that the data differ significantly (*P* < 0.05) as determined by Tukey’s test.

*In silico* analysis of the protein sequence revealed that *Fg*Ars2 (locus ID: FGSG_01106) contains 870 amino acids, and its domain architecture is similar to those of other representative orthologs in some model eukaryotes (Figure 1A). However, *Fg*Ars2 showed a low similarity and identity in protein sequence compared to other representative orthologs; 12–16% in the overall sequence, 18–31% in DUF3546 domain, and 15–39% in ARS2 domain, respectively (Supplementary Figure S1). In addition, the nuclear localization signals predicted by NLStradamus (Ba et al., 2009) displayed two sequences (NLS1, 50 – RRDRRRSRSPAAVDRYEPRPRRG – 72; and NLS2, 134 – MKEEKERARTGRRREPERTRGPEDREKEKA – 163) at its N-terminus, suggesting that *Fg*Ars2 might function as a nuclear protein in *F. graminearum*.

To carry out genetic complementation, the construct containing the *FgARS2* ORF fused with a hygromycin B resistance gene cassette was introduced into protoplasts of the *Fgars2* strain, resulting in DCB101 strains (Supplementary Figure S2). *Fgars2* exhibited reduced radial growth, and the aerial mycelia of *Fgars2* tended to be denser than those of the wild-type and DCB101 strains on both complete medium (CM) and minimal medium (MM) (Figure 1B). The deletion of *FgARS2* also significantly retarded conidial germination compared to the wild-type and DCB101 strains (Figure 1C). In particular, approximately 92 and 88% of conidia in the wild-type and DCB101 strains germinated 5 h after inoculation in CM, respectively, while only about 40% of conidia of the *Fgars2* strains germinated. However, all of conidia from wild-type, *Fgars2*, and DCB101 strains were eventually germinated at 8 h after incubation, indicating their normal viability (Figure 1C).

To decipher the mechanism underlying defective vegetative growth, the DCB102 (*Fgars2 hH1-GFP*) strains were generated by an outcross between the mat1g (*mat1 hH1-GFP*) and *Fgars2* strains (Supplementary Table S1). Microscopic observation revealed that the deletion of *FgARS2* caused a defective phenotype of conidia during nuclear division but did not affect the conidial production, size, or septum number. As a result, 24% of the conidia of DCB102 bore more than two nuclei within a cell, while 10% of these conidia with two nuclei within a cell were scored in the wild-type strain (Figures 1D,E). The mitosis assay further revealed that the lack of *FgARS2* resulted in marked irregularity in nuclear division during germination, while the nuclear division was arrested in both wild-type and *Fgars2* strains when treated with a high concentration of hydroxyurea (HU) (Figure 1E). *Fgars2* strains showed sensitive phenotypes and were significantly inhibited the growth rate against both HU and bleomycin (BLM) which are DNA-damaging agents (Figures 1F,G). The expression level of *FgARS2* in the wild type during HU treatment was significantly higher than that of *FgARS2* without HU treatment (Figure 1H), corroborating the bona fide role of *FgARS2* during DDR in *F. graminearum*. Taken together, these results demonstrated that *FgARS2* is required for vegetative growth and the maintenance of the cell cycle progression in *F. graminearum*.

### *FgARS2* Is Important for Pathogenesis

**FIGURE 2:**
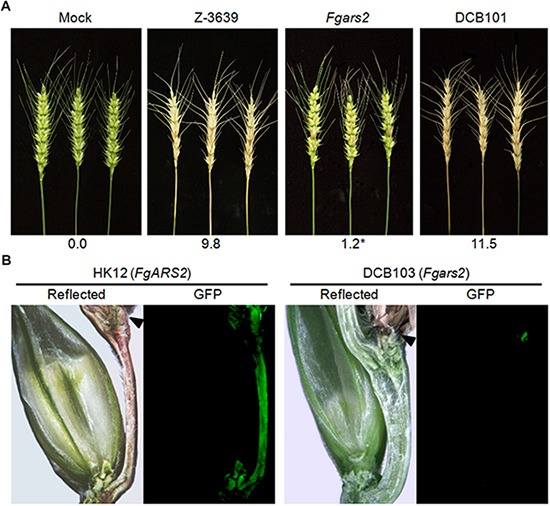
Pathogenesis of *Fgars2* mutants. **(A)** Virulence on wheat heads. The center spikelet of each wheat head was injected with 10 μL of a conidial suspension. The disease index (diseased spikelets per wheat head) is denoted below the picture. The asterisks indicate that the data differ significantly (*P* < 0.05) as determined by Tukey’s test. The pictures were taken 21 days after inoculation. Mock, negative control mock-inoculated with 0.01% Tween 20. **(B)** Micrographs of manually generated sections after infection of wheat. Wheat spikelets were inoculated with conidial suspensions from strains expressing *GFP* in the cytoplasm. Infected wheat heads were longitudinally dissected 6 days after inoculation and examined under a fluorescence microscope. *GFP* fluorescence represented hyphae spreading from the inoculation points. Arrowheads marked the inoculated spikelets. Reflected, reflected light.

Since cell cycle regulation and DDR are closely related to pathogenesis in plant pathogenic fungi, we evaluated the pathogenicity of the *Fgars2* in flowering wheat heads. As expected, the wild-type strain caused typical head blight symptoms of wheat heads (Figure 2A). In contrast, the *Fgars2* mutants were unable to spread from the inoculated spikelet to adjacent spikelets on the heads, leading to a significant decrease in the disease index compared to that in the wild-type strain (Figure 2A). This avirulent feature of the *Fgars2* mutants was fully restored to the wild-type level in DCB101, the *FgARS2*-complemented strain, suggesting the important role of this gene during wheat infection of *F. graminearum*.

We generated an *Fgars2* strain constitutively expressing cytosolic GFP, the DCB103 (*Fgars2 GFP-HYG*), to observe mycelial movement during wheat head infection via an outcross between KM19 (*mat1 GFP-HYG*) and *Fgars2*. The HK12 strain (Son et al., 2011b), *F*. *graminearum* wild-type strain expressing GFP and carrying the *FgARS2* allele, readily colonized the injected spikelet approximately 6 days after inoculation and began to infect adjacent spikelets via rachis nodes (Figure 2B). Although *Fgars2* successfully colonized the injected spikelets as HK12, the hyphae of *Fgars2* could not spread to the neighboring spikelets from those that were inoculated. In addition, hyphal growth via rachis was rarely observed in *Fgars2-*infected plants.

### *FgARS2* Is Involved in Sexual Reproduction

**FIGURE 3:**
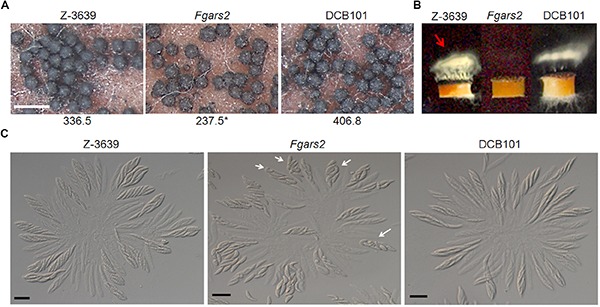
Sexual reproduction of *Fgars2* mutants. **(A)** Sexual development assay. Each strain was inoculated on carrot agar and mock-fertilized. The number of perithecia was counted from 30 mm^2^ of carrot agar. The asterisks indicate that the data differ significantly (*P* < 0.05) as determined by Tukey’s test. The photographs were taken 7 days after sexual induction. Scale bar = 500 μm. **(B)** Forcible ascospore discharge. A semi-circular agar block covered with perithecia was placed on a coverslip. Images were collected 24 h after the assay was initiated. White cloudy material (indicated with a red arrow) represented discharged ascospores. **(C)** Asci rosettes. Imaging was performed 8 days after sexual induction. White arrows indicated asci with defective ascospore delimitation. Scale bar = 20 μm.

The sexual development of the *F. graminearum* strains was observed on 7-day-old sexually induced cultures. All *F. graminearum* strains tested including the *FgARS2* deletion strains produced mature perithecia successfully, however, the perithecium numbers of *Fgars2* were significantly reduced compared to those of the wild type (Figure 3A). The deletion of *FgARS2* also resulted in severely defective forcible ascospore discharge compared to the wild type (Figure 3B). All of these defective phenotypes of the DCB101 strain were restored to the wild-type level during sexual reproduction.

To further unravel the defective phenotypes of *Fgars2* in sexual development, we dissected mature perithecia to observe rosette asci (Figure 3C). The *F. graminearum* wild-type strain typically produced eight spindle-shaped ascospores per ascus. In contrast, some asci of the *Fgars2* mutants that were defective in ascospore discharge contained abnormally shaped ascospores. Further microscopic observation clarified that the abnormally shaped ascospores varied in their morphologies, as some are larger, smaller, and broken (Figure 3C). These data suggest that the defective forcible ascospore discharge of *Fgars2* mutants was derived from abnormalities in ascospore production.

### *FgARS2* Is Not Closely Involved in Small Non-coding RNA Production

**FIGURE 4:**
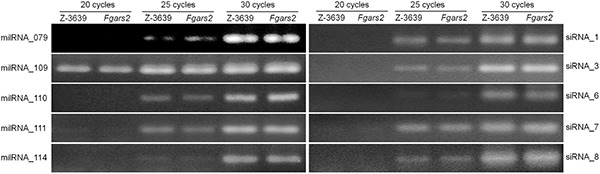
Detection of milRNA and siRNA production. Quantification of milRNA and siRNA candidates was performed using stem-loop RT-PCR assays. Each 100 ng small RNA-enriched RNA samples was used for reverse transcription reactions, and the images were obtained from 20 to 30 cycles of PCR using 3% agarose gels.

*ARS2* and its orthologs have been known to be required for miRNA and siRNA production. Also, our previous study reported that the exonic small interference RNA (ex-siRNA)-mediated RNA interference (RNAi) mechanism fine-tunes the transcriptome during ascospore formation in *F*. *graminearum* (Son et al., 2017). In order to investigate whether *FgARS2* plays an important role for siRNA biogenesis, we first examined the production of ex-siRNA candidates in the wild-type and *Fgars2* strains (Figure 4 and Supplementary Figure S2). Abundances of the siRNAs were easily detectable in both the wild-type and *Fgars2* strains after 20, 25, or 30 cycles of stem-loop PCR (Figure 4). Furthermore, there was an indistinguishable change in the amount of putative ex-siRNAs between the wild-type and *Fgars2* mutant strains (Figure 4).

**FIGURE 5:**
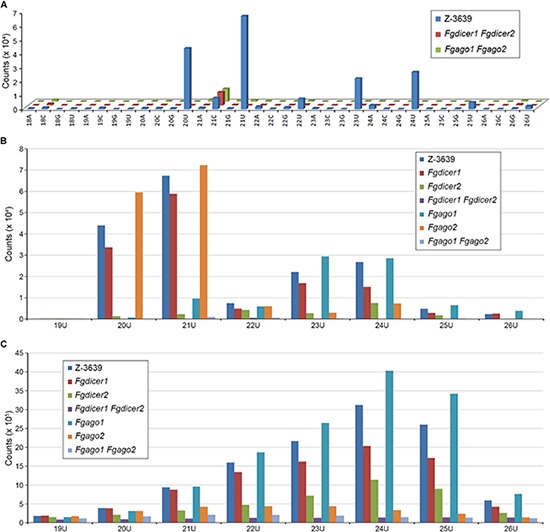
Nucleotide preference of 5′ end and size distribution of sRNAs produced by *F. graminearum* strains. **(A)** Nucleotide preference of the 5′ end and size distribution of milRNA candidates. **(B)** Size distribution of total milRNA candidates with 5′-U. **(C)** Size distribution of total sRNAs with 5′-U.

Although several studies have attempted to characterize microRNA-like RNAs (milRNAs), little is known about the functional milRNA in filamentous fungi (Torres-Martínez and Ruiz-Vázquez, 2017). Considering the conserved function of *ARS2* during the miRNA biogenesis process shown in plants and mammals, we predicted milRNAs in *F. graminearum* based on *in silico* prediction tools that are commonly used for miRNA prediction in model organisms (Figure 5 and Supplementary Figure S3). Using the previously reported miRNA prediction pipeline (Hwang et al., 2013), 1095 miRNA candidates were obtained, reduced to 208 by manual curation, and finally grouped into 109 families in 112 precursors (Supplementary Table S3). No conserved milRNA candidates were identified in a search against the mature miRNAs in the miRBase (Kozomara and Griffiths-Jones, 2013) or even among previously predicted milRNA candidates of *F. oxysporum* or *F. graminearum* (Chen et al., 2014, 2015). Most identified milRNAs were 20 nt or 21 nt long with 5′-U, lacked RNAi (*Fgdicer1 Fgdicer2* and *Fgago1 Fgago2*) greatly abolished milRNA production (Figure 5A). Intriguingly, the predicted milRNAs that were 20 nt and 21 nt long were exclusively produced by *Fgdicer2* and *Fgago1*, while both *Fgdicers* and *Fgago2* were important for global sRNA production in *F. graminearum* (Figures 5B,C).

We further determined the amounts of milRNAs candidates in the wild-type and *Fgars2* strains. Similar to the siRNAs, the amounts of putative milRNAs were unaffected by the disruption of *FgARS2* compared to the wild type (Figure 4). Taken together, our results suggest that *FgARS2* is not closely involved in the milRNA and siRNA production in *F. graminearum*.

### Subcellular Localization of *Fg*Ars2

**FIGURE 6:**
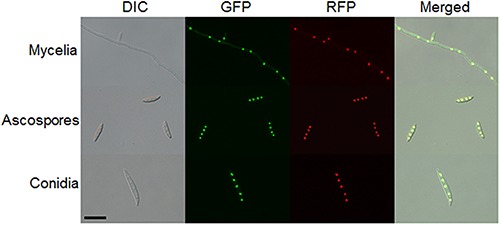
Subcellular localization of *Fg*Ars2. A DCB104r strain containing *Fg*Ars2-GFP and hH1-RFP was grown on CM (mycelia), carrot agar medium (ascospores), or YMA (conidia) for microscopic observation. DIC, differential interference contrast. Scale bar = 10 μm.

Because two nuclear localization signal sequences were observed at the *Fg*Ars2 N-terminus, we examined the subcellular localization of *Fg*Ars2, which might provide insights to understand its biological functions. To achieve this, we generated strains overexpressing the *FgARS2* gene fused with *GFP*, resulting in DCB104 strains (Supplementary Figure S4). It is noteworthy that the DCB104 strains possessed indistinguishable phenotypes compared to the wild-type strain (data not shown). To confirm the nuclear localization of *Fg*Ars2-GFP, the DCB104r (*FgARS2:P_*EF*1*a*_-GFP-FgARS2-GEN*; *hH1-RFP-GEN*) strains were generated via outcrossing of the mat1r (Son et al., 2011a) and DCB104 strains. Localization was observed in cultures grown on CM (for hyphae), YMA (for conidia), and carrot agar (for ascospores). *Fg*Ars2-GFP in the DCB104r strains co-localized with hH1-RFP (Figure 6) in all of the observed developmental stages, validating the distinct nuclear localization of *Fg*Ars2.

### *Fg*Ars2 and Cap Binding Complex Proteins Are Important for Pathogenesis

Since the Ars2 protein physically interacts with the CBC proteins in model organisms, we questioned whether the interaction is still conserved in *F. graminearum*. In model eukaryotes, the CBC consists of 20 and 80 kDa proteins, denominated Cbp20 (cap-binding protein 20) and Cbp80, respectively (Gonatopoulos-Pournatzis and Cowling, 2014). The plant Cbp20/80 protein sequences were utilized to identify CBC orthologs in *F. graminearum* using the BLAST algorithm^1^. There is only one orthologous gene for each CBC subunit found in the *F. graminearum* genome, assigned as *FgCBP20* (locus ID: FGSG_05488) and *80* (locus ID: FGSG_07328), respectively. Accordingly, *Fg*Cbp20 encoded a 173-amino-acid (aa) protein, and *Fg*Cbp80 was a long protein with 798 aa. We used the yeast two-hybrid system to validate the interaction between *Fg*Cbp20, *Fg*Cbp80, and *Fg*Ars2.

**FIGURE 7:**
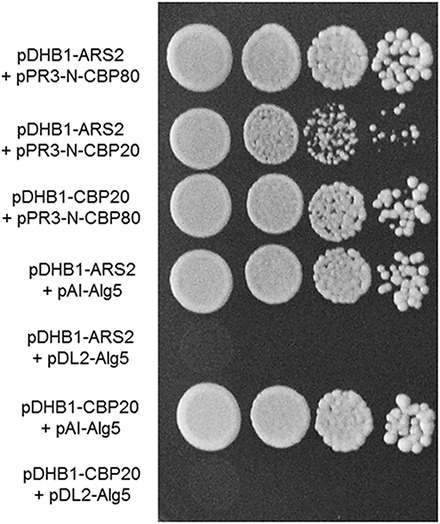
Yeast two-hybrid analysis of the interaction between *Fg*Ars2 and cap binding complex. The pairs of plasmids pDHB1-ARS2/pAI-Alg5 and pDHB1-ARS2/pDL2-Alg5 as well as pDHB1-CBP20/pAI-Alg5 and pDHB1-CBP20/pDL2-Alg5 served as positive and negative controls, respectively. The growth of transformed yeast was assayed on synthetic dextrose medium lacking His, Leu, Trp, and Ade. Columns in each panel display the serial decimal dilution.

Our results of yeast two-hybrid assay revealed that *Fg*Cbp80 physically interacted with *Fg*Cbp20, suggesting that they form the heterodimeric cap-binding complex (Figure 7). *Fg*Ars2 interacted with both *Fg*Cbp80 and *Fg*Cbp20, although the strength of the *Fg*Ars2-*Fg*Cbp20 interaction was weaker than that of *Fg*Ars2-*Fg*Cbp80, which is consistent with those in plants and mammals. These results suggest that the *Fg*Ars2-*Fg*Cbp80-*Fg*Cbp20 complex may involve some conserved functions or might play novel functions in filamentous fungi, including *F. graminearum*.

**FIGURE 8:**
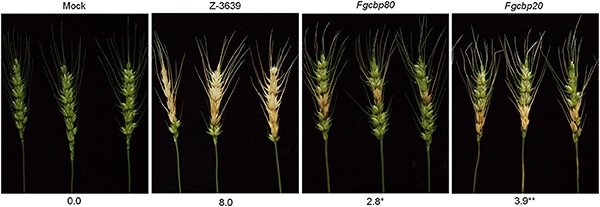
Virulence of *F. graminearum* cap binding complex mutants on wheat heads. The center spikelet of each wheat head was injected with 10 μL of a conidial suspension. The disease index (diseased spikelets per wheat head) is denoted below the picture. The asterisks indicate that the data differ significantly (*P* < 0.05) as determined by Tukey’s test. The pictures were taken 21 days after inoculation. Mock, negative control mock-inoculated with 0.01% Tween 20.

To further explore the genetic and biological functions of the module *Fg*Ars2-*Fg*Cbp80-*Fg*Cbp20 in *F. graminearum*, we constructed and characterized the single deletion mutants of *FgCBP80* and *FgCBP20* in detail (Supplementary Table S1 and Supplementary Figure S5). Similar to *FgARS2*, *FgCBP80* and *FgCBP20* were not essential for somatic growth in this fungus. In addition, single deletions of both *FgCBP80* and *FgCBP20* produced morphologically indistinguishable phenotypes compared to the wild-type strain (data not shown). Intriguingly, both the *Fgcbp80* and *Fgcbp20* mutants drastically attenuated pathogenicity on wheat heads, although these defects were milder than that of *Fgars2* (Figure 8). These results strongly fulfilled the hypothesis that CBC proteins are required for the pathogenesis of *F. graminearum* and suggested that the robust physical interaction of *Fg*Ars2 with *Fg*Cbp80-*Fg*Cbp20 complex might be specifically required for virulence in this fungus.

## Discussion

We identified sixteen putative TFs involved in DDR in our previous study and further demonstrated that the cell cycle regulation and DDR were closely related to sexual reproduction and virulence in *F. graminearum* (Min et al., 2014; Son et al., 2016). In this study, we uncovered the novel cellular function of the *ARS2* ortholog in DDR as well as pathogenesis in the plant pathogenic fungus *F. graminearum*. Our results showed its diverse roles in the fungal developmental processes, while the molecular function of *Fg*Ars2 in the sRNA production did not conserved in *F. graminearum*. In addition, we provided genetic evidences demonstrating that *Fg*Ars2 physically interacts with CBC proteins to form a robust CBCA complex, and the stability of this tertiary complex governs fungal pathogenesis.

ARS2 takes part in global post transcriptional regulation and therefore ARS2 orthologs have been shown to be essential in fission yeast, plants, insects, and mammals (Golling et al., 2002; Oh et al., 2003; Amsterdam et al., 2004; Lobbes et al., 2006; Wilson et al., 2008; Kim et al., 2010). In contrast, *FgARS2* deletion mutants were viable in *F. graminearum* (Son et al., 2011c). Our *in silico* analysis indicated that *Fg*Ars2 preserved the functional domains, and the deletion of *FgARS2* resulted in pleiotropic defects in the fungal developmental processes of *F. graminearum* (Figures 1, 3). *HsARS2* knockdown disrupted the replication-dependent histone mRNA processing and caused a reduction in histone expression, leading to disruption of the cell cycle (Kiriyama et al., 2009; Gruber et al., 2012) and *AtSE* has also been shown to control leaf development, meristem activity, and inflorescence architecture in *A. thaliana* (Machida et al., 2011). Combined, Ars2 likely plays conserved roles in cell cycle progression and proliferation from mammals, insects, and plants to fungi and other lower eukaryotes.

The growth rate of *Fgars2* appears to be sensitive during treatment with DNA-damaging agents, and the transcript level of *FgARS2* is increased after treatment with DNA-damaging agent (Son et al., 2016). Our results revealed that *Fgars2* was irregular in nuclear division, and its conidial germination was significantly retarded, presumably due to its hypersensitive phenotypes during DNA-damage stress (Figure 1). Similarly, myogenic cells deficient in *HsARS2* resulted in a slow cell cycle and spent an increased time in the S phase (O’sullivan et al., 2015). Thus, our findings further deciphered the important role of Ars2 in the proper mitotic cell division and DDR in filamentous fungi and possibly in other eukaryotes.

The physical interaction between ARS2 and CBC proteins through the 7-methylguanosine cap structure of nuclear RNA polymerase II transcripts has been extensively documented (Laubinger et al., 2008; Gruber et al., 2009; Sabin et al., 2009). In addition, the vast majority of ARS2 functions involved in its interactions with CBP20/80 is conserved in model eukaryotes (O’sullivan and Howard, 2016). In our study, *Fg*Ars2 physically established a robust interaction with both *Fg*Cbp80 and *Fg*Cbp20 proteins, indicating that this complex structure is evolutionally conserved in eukaryotes. Similarly, *Nc*Cbp20 and *Nc*Cbp80 directly interact to form the CBC, and this heterodimeric complex is predominantly nuclear in *N. crassa* (Decker et al., 2017). Genetic observation also showed that *FgCBP20* and *80* were not essential for somatic growth as previously reported in *N. crassa* (Decker et al., 2017). While the disruption of *NcCBC* causes a drastic reduction in ascospore production, the single deletion of *FgCBP20* or *FgCBP80* resulted in morphologically indistinguishable phenotypes compared to the wild-type strain. Instead, our results clarified that as is the case with *Fgars2*, both *Fgcbp20* and *Fgcbp80* mutants attenuated their virulence on wheat heads, suggesting a role of the CBCA theme in fungal pathogenesis.

The *Fgars2* mutants produced reduced amounts of trichothecene, however, they grew similarly under oxidative stress condition compared to the wild-type (Son et al., 2011c). Moreover, *Fgars2* strains showed sensitive phenotypes against several DNA-damaging agents, HU and BLM (Figures 1F,G). Because HU inhibits DNA replication by inhibiting ribonucleotide reductase and BLM induces DNA double-strand breaks, we suspected that FgArs2 is involved in the DDR in *F. graminearum* (Ehrenberg and Reichard, 1972; Stubbe and Kozarich, 1987). Given that plant defenses can also elicit damages to DNA, there should be a link between virulence and DDR. Collectively, based on our achieved data, the attenuated virulence of *Fgars2* mutant is likely regulated by combination of different factors, including reduced trichothecene production, DDR defects, and dysregulation of CBCA theme. Further investigations would be necessary to decode this sophisticated interaction.

In last years, studies on model eukaryotes revealed that *ARS2* is required for proper miRNA biogenesis. AtSE acts as a scaffold to stimulate the efficiency and accuracy of pri-miRNA processing by the *At*DCL-1/*At*HYL-1 complex (Dong et al., 2008; Machida et al., 2011); therefore, the disruption of *AtSE* resulted in decreased levels of mature miRNAs and increased levels of pri-miRNA transcripts (Grigg et al., 2005; Lobbes et al., 2006; Yang et al., 2006). In mammalian systems, the decreased levels of mature miR-21, let-7 miRNAs, as well as primary miR-21 transcripts, were the consequence of *HsARS2* depletion (Gruber et al., 2009). Both *DmARS2* and *DmCBC* are required for miRNA- and siRNA-mediated silencing as well as antiviral defense in *D. melanogaster* (Sabin et al., 2009).

Most eukaryotes except some yeast species possess conserved RNAi mechanism. While higher eukaryotes such as plants and animals have evolved a miRNA-mediated RNAi as an important post-transcriptional gene regulation mechanism, endogenous ex-siRNA-mediated transcriptional regulation play a crucial role in fungal development and stress responses in lower eukaryotic fungi such as a basal fungus *Mucor circinelloides* (Torres-Martínez and Ruiz-Vázquez, 2016). *F. graminearum* only utilize ex-siRNA-mediated RNAi during late stages of sexual development, but not for other developmental stages or genome defenses (Son et al., 2017). This study uncovered that *Fg*Ars2 is critically required for ascosporogenesis but dispensable for the production of putative milRNAs and siRNAs. In line with this, some functions of Ars2, such as the role for miRNA or siRNA production, seem to have been lost in *F. graminearum*. However, Ars2-dependent cellular processes have been evolved to govern sexual reproduction, DDR, and virulence in this fungus. In particular, formation of Ars2-CBC complex was revealed to be important for fungal virulence.

## Conclusion

Our data proved that *FgARS2* is required for diverse roles in DDR, fungal progressions, and pathogenesis, but not for sRNA production in *F. graminearum*. In addition, *Fg*Ars2 physically interacted with the CBC, and this robust theme, together with trichothecene and DDR, seemed to be essential factors governing fungal pathogenesis in *F. graminearum*. Taken together, our study provides new insights into the diverse roles of *ARS2* ortholog in pathogenic fungi during fungal development and pathogenesis.

## Data Availability Statement

All datasets generated for this study are included in the manuscript/Supplementary Files.

## Author Contribusions

D-CB, J-EK, Y-WL, J-AS, and HS conceived and designed the experiments. D-CB, J-EK, JS, GC, and HS performed the experiments. D-CB, J-EK, JL, and J-AS analyzed the data. GC, Y-WL, J-AS, and HS contributed the reagents, materials, and analysis tools. D-CB, J-EK, JS, Y-WL, J-AS, and HS wrote the manuscript. All authors read and approved the final version of manuscript.

## Conflict of Interest

The authors declare that the research was conducted in the absence of any commercial or financial relationships that could be construed as a potential conflict of interest.

## Footnotes

1 http://fungidb.org
