# Supplementary material for: ARS2 Plays Diverse Roles in DNA Damage Response, Fungal Development, and Pathogenesis in the Plant Pathogenic Fungus Fusarium graminearum
Source: Front Microbiol. 2019 Oct 15;10:2326. doi: 10.3389/fmicb.2019.02326 (PMC6803386; doi:10.3389/fmicb.2019.02326)
Supplement: Supplementary file 1 [file Data_Sheet_1.docx]

***Supplementary Materials***

***ARS2* plays diverse roles in DNA damage response, fungal development, and pathogenesis in the plant pathogenic fungus *Fusarium graminearum***

**Duc-Cuong Bui^1, 2†^, Jung-Eun Kim^1^, Jiyoung Shin^1^, Jae Yun Lim^2^, Gyung Ja Choi^3^, Yin-Won Lee^1^, Jeong-Ah Seo^2*^, and Hokyoung Son^1*^**

*^1^ Department of Agricultural Biotechnology and Research Institute of Agriculture and Life Sciences, Seoul National University, Seoul, South Korea*

*^2^ School of Systems Biomedical Science, Soongsil University, Seoul, South Korea*

*^3^ Therapeutic & Biotechnology Division, Center for Eco-friendly New Materials, Korea Research Institute of Chemical Technology, Daejeon, South Korea*

**^*^Correspondences:**

*Jeong-Ah Seo*

*sja815@ssu.ac.kr*

*Hokyoung Son*

*hogongi7@snu.ac.kr*

^†^Present address:

*Department of Pathology and Microbiology,* [*University of Nebraska Medical Center*](https://scholar.google.co.kr/citations?view_op=view_org&hl=ko&org=17229611625391662695)*, Omaha, NE, United States*

**Table S1. *F. graminearum* strains used in this study.**

| **Strain** | **Genotype** | **Reference, source, or parent strains** |
| --- | --- | --- |
| Z-3639 | *Fusarium graminearum* wild-type | Bowden and Leslie, 1999 |
| hH1-GFP | *hH1::hH1-GFP-HYG* | Hong et al., 2010 |
| mat1g | Δ*mat1-1::GEN hH1::hH1-GFP-HYG* | Hong et al., 2010 |
| mat1r | Δ*mat1-1::GEN hH1::hH1-RFP-HYG* | Son et al., 2011a |
| HK12 | *GFP-HYG* (GFP constitutive expresser in cytosol) | Son et al., 2011b |
| KM19 | Δ*mat1-1-1::GEN GFP-HYG* | Min et al., 2012 |
| *Fgars2* | Δ*Fgars2::GEN* | Son et al., 2011c |
| DCB101 | Δ*Fgars2::FgARS2-HYG* | This study |
| DCB102 | Δ*Fgars2::GEN hH1-GFP-HYG* | mat1g × *Fgars2* |
| DCB103 | Δ*Fgars2::GEN GFP-HYG* | KM19 × *Fgars2* |
| DCB104 | Δ*Fgars2::GEN- P_EF1a_ -GFP-FgARS2* | This study |
| DCB104r | Δ*Fgars2::GEN- P_EF1a_ -GFP-FgARS2 hH1::hH1-RFP-GEN* | This study |
| *Fgcbp80* | Δ*Fgcbp80::GEN* | This study |
| *Fgcbp20* | Δ*Fgcbp20::GEN* | This study |

**Table S2. Primers used in this study.**

| **Primer** | **Sequence (5ʹ→3ʹ)** | **Description** |
| --- | --- | --- |
| FgARS2-5F com | CAACCAACCAACTTAGCCTCTTCAAC | Forward, forward nest, and reverse primers for amplification of *FgARS2* with tail (lowercase) for the hygromycin B resistancegene cassette fusion from pBCATPH |
| FgARS2-5N com | TACTCTCTTTTCCCCTGATCCCTCTT |  |
| FgARS2-3N com | tgagacaaatggtgttcaggatctcAGAGACTGACCTGGAGGCATAAGAAAT |  |
| FgARS2-5F OEG | GGGAAGTGATTTGGCGAGATGTAA | Forward and reverse primers for amplification of 5ʹ-flanking region of *FgARS2* with tail (lowercase) for *GFP* tagging overexpression through *EF* promoter |
| FgARS2-5R OEG | gatagtggaaaccgacgccccTGTTGGTATCGTTGCCAGCGAC |  |
| FgARS2-3F OEG | cggcatggacgagctgtacaagATGGACTCGTACACATCTTTTCGGG | Forward and reverse primers for amplification of *FgARS2* ORF as 3ʹ-flanking region with tail (lowercase) for *GFP* tagging overexpression of *FgARS2* through *EF* promoter |
| FgARS2-3R OEG | CAGAGAGAGCCACTTGAAACCACC |  |
| FgARS2-5N OEG | CAACCAACCAACTTAGCCTCTTCAAC | Forward and reverse nest primers for third fusion PCR for *GFP* tagging overexpression of *FgARS2* through *EF* promoter |
| FgARS2-3N OEG | GCTCCCACTGGCTGTAGGCAC |  |
| Neo-for new | GGGGCGTCGGTTTCCACTATC | Forward and reverse primers for amplification of *GEN-EF-GFP* from pSKGEN vector |
| eGFP-P1 | CTTGTACAGCTCGTCCATGCCG |  |
| Gen-For | CGACAGAAGATGATATTGAAGG | Forward and reverse primers for amplification of the geneticin cassette from the pII99 vector |
| Gen-Rev | CTCTAAACAAGTGTACCTGTGC |  |
| Gen-G2 | GCAATATCACGGGTAGCCAACG | Forward and reverse nest primers for split marker amplification of geneticin resistance gene cassette |
| Gen-G3 | GGGAAGGGACTGGCTGCTATTG |  |
| pBCATPH-comp 5ʹFor | GTGAGCGGATAACAATTTCACACAG | Forward and reverse primers for amplification of hygromycin B resistance gene cassette from pBCATPH |
| pBCATPH-comp 3ʹRev | GAGATCCTGAACACCATTTGTCTCA |  |
| pIGPAPA-H2 | TCGCTCCAGTCAATGACCGC | Forward and reverse nest primers for split marker amplication of hygromycin B resistance gene cassette from pBCATPH |
| pU,pBC-H3 | CGTTATGTTTATCGGCACTTTGC |  |
| FgARS2-5F | TGCCTTTTCTGGATGATGATTGGT | Forward and reverse primers for amplification of 5ʹ-flanking region of *FgARS2* with tail (lowercase) for the geneticin resistance gene cassette fusion |
| FgARS2-5R | gcacaggtacacttgtttagagGACGCGGCATGAAAGAAGGTTG |  |
| FgARS2-3F | ccttcaatatcatcttctgtcgTGGACCAGCCGTTTATCAACAGAAT | Forward and reverse primers for amplification of 3ʹ-flanking region of *FgARS2* with tail (lowercase) for the geneticin resistance gene cassette fusion |
| FgARS2-3R | GCGTAGATGTGCCAGAAGGTGTC |  |
| FgARS2-5N | CACGCAACAAAGGATATGAACTGATT | Forward and reverse nest primers for third fusion PCR for amplification of the *FgARS2* deletion construct |
| FgARS2-3N | CTTCTCCTCGTCTGACCCATCTGA |  |
| ARS2-rt-5F | TGTCCGATCCAAACCCCAGTAA | For realtime-PCR of *FgARS2* |
| ARS2-rt-5R | CCAACGTGGCAGACGAAATCA |  |
| UBH1-rt-F | GTTCTCGAGGCCAGCAAAAAGTCA | For realtime-PCR of *FgUBH1* |
| UBH1-rt- R | CGAATCGCCGTTAGGGGTGTCTG |  |
| FgmilRNA-079 RT | GTCGTATCCAGTGCAGGGTCCGAGGTATTCGCACTGGATACGACATCTCT | RT stem-loop primer for RT-PCR of FgmilRNA-079 |
| FgmilRNA-079 F | ACTAACGGGCTCATTCGATCTTTGT | Forward primer for RT-PCR of FgmilRNA-079 |
| FgmilRNA-109 RT | GTCGTATCCAGTGCAGGGTCCGAGGTATTCGCACTGGATACGACCAACCA | RT stem-loop primer for RT-PCR of FgmilRNA-109 |
| FgmilRNA-109 F | CAAGCCTCCGTGATAAATTGGTGT | Forward primer for RT-PCR of FgmilRNA-109 |
| FgmilRNA-110 RT | GTCGTATCCAGTGCAGGGTCCGAGGTATTCGCACTGGATACGACCTTCAT | RT stem-loop primer for RT-PCR of FgmilRNA-110 |
| FgmilRNA-110 F | TGCCCGTTCTGGCATTGTTGTG | Forward primer for RT-PCR of FgmilRNA-110 |
| FgmilRNA-111 RT | GTCGTATCCAGTGCAGGGTCCGAGGTATTCGCACTGGATACGACAATCTT | RT stem-loop primer for RT-PCR of FgmilRNA-111 |
| FgmilRNA-111 F | CATGCTCAGAGCGGTGATTGATC | Forward primer for RT-PCR of FgmilRNA-111 |
| FgmilRNA-114 RT | GTCGTATCCAGTGCAGGGTCCGAGGTATTCGCACTGGATACGACTCCAAG | RT stem-loop primer for RT-PCR of FgmilRNA-114 |
| FgmilRNA-114 F | CCATGGCCTGTTGACAGAGAATCT | Forward primer for RT-PCR of FgmilRNA-114 |
| siRNA-R | GTGCAGGGTCCGAGGT | Common reverse primer for RT-PCR of siRNA |
| FgsiRNA-1 RT | GTCGTATCCAGTGCAGGGTCCGAGGTATTCGCACTGGATACGACGGGCAA | RT stem-loop primer for RT-PCR of FgmilRNA-110 |
| FgsiRNA-1 F | TCGCCCTATCGATTGTAGAAAGCC | Forward primer for RT-PCR of FgmilRNA-110 |
| FgsiRNA-3 RT | GTCGTATCCAGTGCAGGGTCCGAGGTATTCGCACTGGATACGACTACGCA | RT stem-loop primer for RT-PCR of FgsiRNA-3 |
| FgsiRNA-3 F | GTCTCAAGCAAGAGTTCTGCGG | Forward primer for RT-PCR of FgsiRNA-3 |
| FgsiRNA-6 RT | GTCGTATCCAGTGCAGGGTCCGAGGTATTCGCACTGGATACGACTCTGAA | RT stem-loop primer for RT-PCR of FgsiRNA-6 |
| FgsiRNA-6 F | GTACCCGGGTCGTCAGTGA | Forward primer for RT-PCR of FgsiRNA-6 |
| FgsiRNA-7 RT | GTCGTATCCAGTGCAGGGTCCGAGGTATTCGCACTGGATACGACAAAGAG | RT stem-loop primer for RT-PCR of FgsiRNA-7 |
| FgsiRNA-7 F | GGGCGCCAGAGGTCGA | Forward primer for RT-PCR of FgsiRNA-7 |
| FgsiRNA-8 RT | GTCGTATCCAGTGCAGGGTCCGAGGTATTCGCACTGGATACGACTATGAA | RT stem-loop primer for RT-PCR of FgsiRNA-8 |
| FgsiRNA-8 F | TCCTCGGTCTGCCGCAAG | Forward primer for RT-PCR of FgsiRNA-8 |
| FgCBP20-bait/prey-F *Sfi*I | aacgcagagaggccattacggccATGCGACCTGGCGTCAGAAGTA | Forward and reverse primer for amplification of cDNA of *FgCBP20* |
| FgCBP20-bait/prey-R *Sfi*I | aacgcagagaggccgaggcggccgCTTGCCTTCAGCGTACTCGTCTC |  |
| FgCBP80-prey-F *Sfi*I | aacgcagagaggccattacggccATGGCCGACTACGATAGACGACC | Forward and reverse primer for amplification of cDNA of *FgCBP80* |
| FgCBP80-prey-R *Sfi*I | aacgcagagaggccgaggcggccgATTCTCCGCTCCGTCAGTTGC |  |
| FgARS2-bait-F *Sfi*I | aacgcagagaggccattacggccATGGACTCGTACACATCTTTTCGGG | Forward and reverse primer for amplification of cDNA of *FgARS2* |
| FgARS2-bait-R *Sfi*I | aacgcagagaggccgaggcggccaaGCTTCCGCCACCAGAGACTTG |  |
| FgCBP20-5F | GGACAGCAGACAGATAACAGCGAGA | Forward and reverse primers for amplification of 5ʹ-flanking region of *FgCBP20* with tail (lowercase) for the geneticin resistance gene cassette fusion |
| FgCBP20-5R | gcacaggtacacttgtttagagCCTGTGGCTCTATCGCTGGGTT |  |
| FgCBP20-3F | ccttcaatatcatcttctgtcgGTCATATAAGACTGCCGCCAAAGC | Forward and reverse primers for amplification of 3ʹ-flanking region of *FgCBP20* with tail (lowercase) for the geneticin resistance gene cassette fusion |
| FgCBP20-3R | ACGACCTTGCGAAATGAATACCAC |  |
| FgCBP20-5N | CCTCGGGGTCATCCTCAATACTG | Forward and reverse nest primers for third fusion PCR for amplification of the *FgCBP20* deletion construct |
| FgCBP20-3N | CCATATTGGCACGGTAGAAAGGACT |  |
| FgCBP80-5F | CATCTCCATCAATCACCCTTAGCAAT | Forward and reverse primers for amplification of 5ʹ-flanking region of *FgCBP80* with tail (lowercase) for the geneticin resistance gene cassette fusion |
| FgCBP80-5R | gcacaggtacacttgtttagagATCCATGGCAATGCGTCTGTG |  |
| FgCBP80-3F | ccttcaatatcatcttctgtcgCATGAGGCGCTACTAGGGGAAAG | Forward and reverse primers for amplification of 3ʹ-flanking region of *FgCBP80* with tail (lowercase) for the geneticin resistance gene cassette fusion |
| FgCBP80-3R | GATTTCGGAAGCGCCAAGATTT |  |
| FgCBP80-5N | CCATCCGATTTCCCATTTCATTTAG | Forward and reverse nest primers for third fusion PCR for amplification of the *FgCBP80* deletion construct |
| FgCBP80-3N | GGAGATGATGGCGAAACTGGATT |  |

**
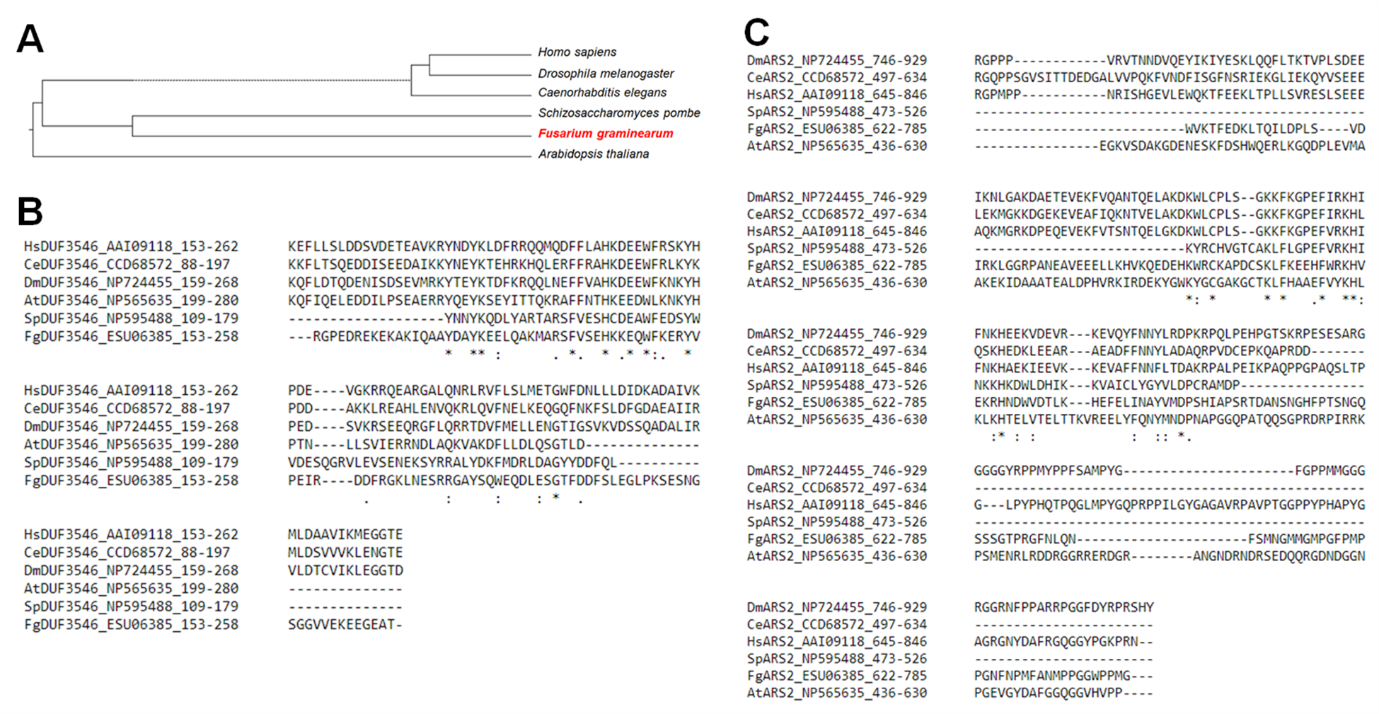
**

**Supplementary Figure 1 | In silico analysis of the arsenite-resistance protein 2 (Ars2).** (A) Phylogenetic tree of orthologs of the Ars2 from representative species constructed using entire amino acid sequences comparison. Alignment analysis of the evolutionary conservation Ars2 protein in specific domains, including DUF3546 domain (B) and ARS2 domain (C), from representative species. The amino acid sequences of Ars2 orthologs *Arabidopsis thaliana* (NP_565635), *Homo sapiens* (AAI09118), *Drosophila melanogaster* (NP_724455), *Caenorhabditis elegans* (CCD68572.1), *Schizosaccharomyces pombe* (NP_595488), *F. graminearum* (Gene ID: FGSG_01106) are shown.


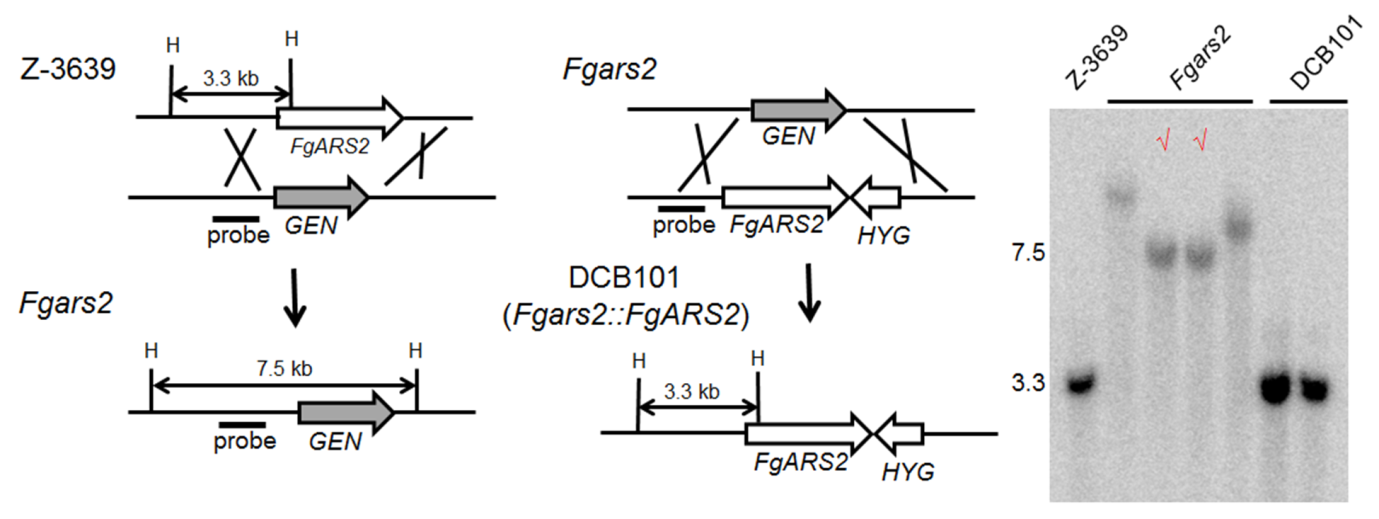


**Supplementary Figure 2 | Targeted deletion and complementation of *FgARS2*.** Left and middle panels: Targeted deletion and complementation of *FgARS2*. Right panel: Southern blot analysis confirming genetic construct. The 5ʹ-flanking regions (black bars) of the *FgARS2* were used as probes for hybridization. The sizes of DNA products (kb) were indicated to the left of the blot. Z-3639, *F. graminearum* wild-type strain Z-3639; *Fgars2*, *FgARS2* deletion mutant; DCB101, *Fgars2*-derived strain complemented with *FgARS2*; H, *Hind*III; *GEN*, geneticin resistance gene cassette; *HYG*, hygromycin B resistance gene cassette.


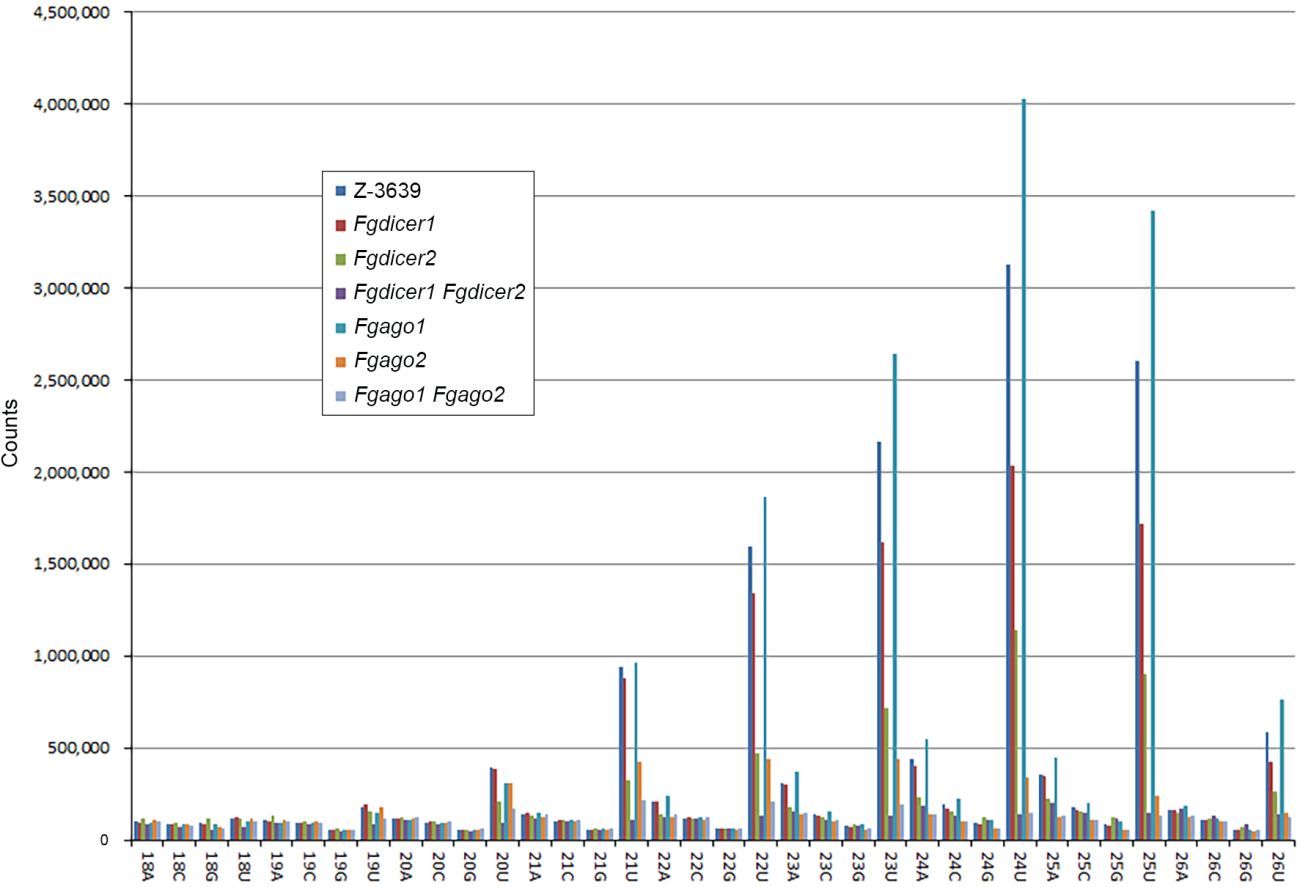


**Supplementary Figure 3 | Nucleotide preference of 5′ end and size distribution of sRNAs produced by *F. graminearum* strains.**


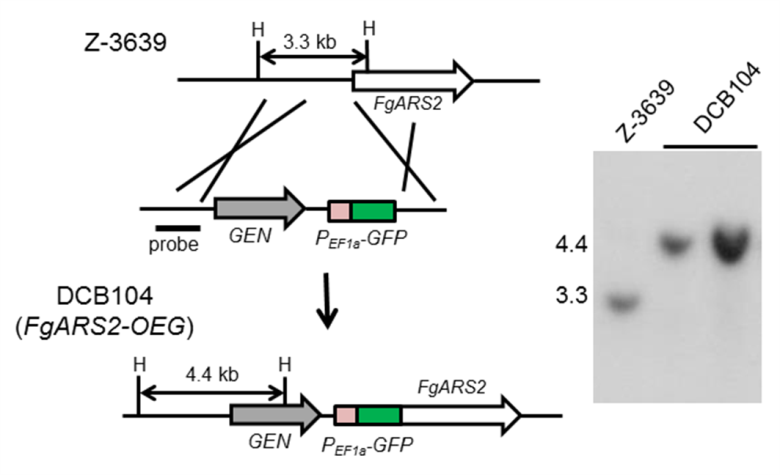


**Supplementary Figure 4 | Strategy for *GFP* fusion with the *EF1a* promoter.** Left panel: *FgARS2* was fused with green fluorescent protein (*GFP*). Right panel: Southern blot analysis confirming genetic construct. The 5ʹ-flanking regions (black bars) of the *FgARS2* ORF were used as probes for hybridization. The sizes of DNA products (kb) were indicated to the left of the blot. Z-3639, *F. graminearum* wild-type strain Z-3639; DCB104, overexpressing *FgARS2* gene fused with *GFP* strain; H, HindIII; *GEN*, geneticin resistance gene cassette.


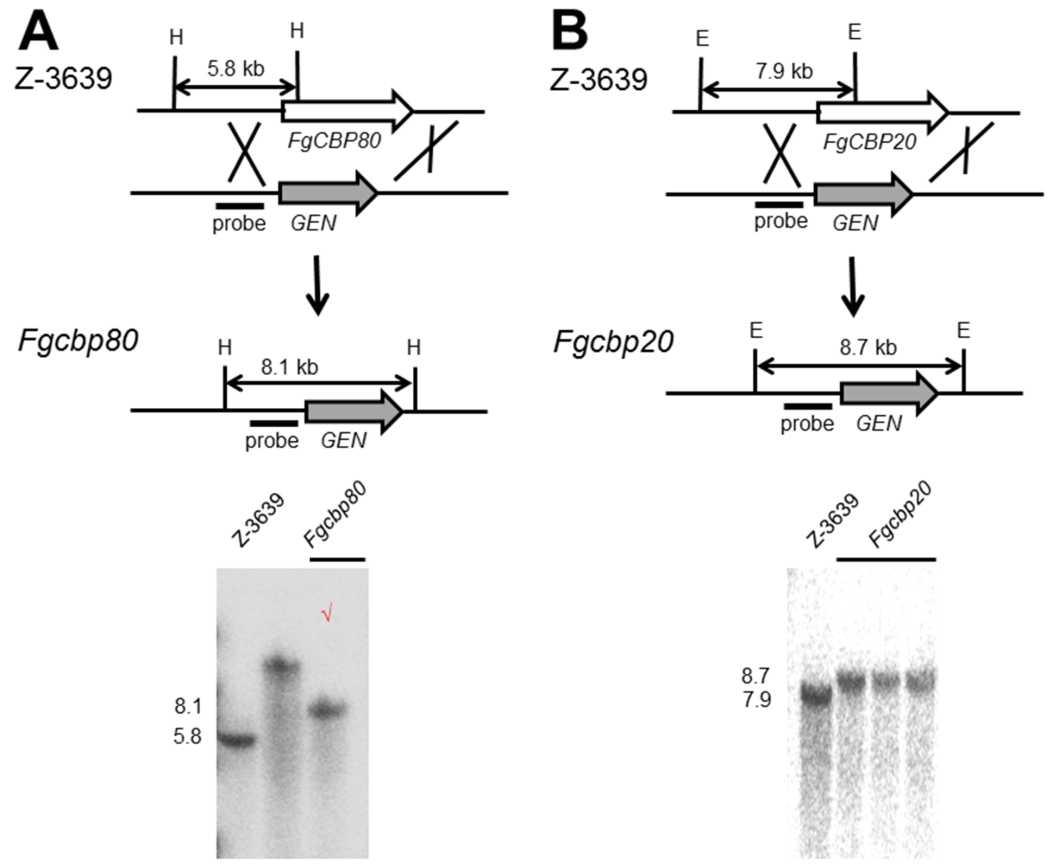


**Supplementary Figure 5 | Targeted deletion strategies of cap binding protein subunit 80** **(A) and 20 (B)**. Top and middle panels: Targeted deletion strategies. Bottom panel: Southern blot analysis confirming genetic construct. The 5ʹ-flanking regions (black bars) of the *FgCBP80* or *FgCBP20* ORF were used as probes for hybridization. The sizes of DNA products (kb) were indicated to the left of the blot. Z-3639, *F. graminearum* wild-type strain Z-3639; *Fgcbp80*, *FgCBP80* deletion mutant; *Fgcbp20*, *FgCBP20* deletion mutant; H, *Hind*III; E, *EcoR*V; *GEN*, geneticin resistance gene cassette.
